# Molecular Dynamics Simulation: Tendency for CO_2_ Adsorption in Amphiphilic Cellulose-Derived Interpenetrating Network Gels

**DOI:** 10.3390/gels12060537

**Published:** 2026-06-15

**Authors:** Funsho Afolabi, Zulhelmi Amir, Ahmed Halilu, Muhamad Fazly Abdul Patah, Eugene N. Ngouangna, Akorede O. Joledo, Pearl I. Murungi

**Affiliations:** 1 Department of Chemical Engineering, Universiti Malaya, Kuala Lumpur 50603, Malaysia; fazly.abdulpatah@um.edu.my; 2 Centre for Integrative Petroleum Research, King Fahd University of Petroleum & Minerals, Dhahran 31261, Saudi Arabia; 3 Department of Petroleum Engineering, University of Ibadan, Ibadan 200005, Nigeria; ao.joledo@ui.edu.ng; 4 Department of Petroleum Engineering, Universiti Teknologi PETRONAS, Seri Iskandar 32610, Malaysia

**Keywords:** CO_2_, interpenetrating network, adsorption, geosequestration, molecular dynamics simulation, composite gel

## Abstract

The subject of CO_2_ subsurface storage security has never been more critical, and there is a need to explore the injection of functional materials that are capable of providing both conformance control and in situ CO_2_ adsorption, thereby improving overall formation storage integrity. Herein, a molecular dynamics simulation method was used to investigate the adsorptive tendency of two variants of interpenetrating network (IPN) composite materials comprising amine-stabilized hydrophobically modified cellulose sulphates and methylene bisacrylamide crosslinked polyacrylamide. Using the COMPASS III force field and Metropolis Monte Carlo, the diffusivity and adsorption isotherms for CO_2_ were determined in the IPN gels, respectively. The results indicate that the two interpenetrating networks D-I-AM-MBA-G-Cl and D-II-AM-MBA-G-Cl demonstrated reasonable CO_2_ adsorption. In saline conditions, the adsorption was further enhanced with diffusion coefficients of 4.87 × 10^−4^ cm^2^/s and 2 × 10^−6^ cm^2^/s. The adsorption isotherm of D-I-AM-MBA-G-Cl closely fits the Sips equation, with a regression coefficient of 0.9996, while that of D-II-AM-MBA-G-Cl follows the Temkin isotherm with an R^2^ value of 0.9885. This study revealed that carefully designed plugging agents with strong CO_2_ adsorption tendencies can aid in the improvement of the geosequestration integrity of subsurface formations.

## 1. Introduction

The subsurface storage of CO_2_ has increasingly become a focal point in contemporary oil and gas practices due to its critical role in climate stabilization and the mitigation of further global warming. This growing emphasis is supported by decades of operational experience in cyclic CO_2_ injection and production for enhanced oil recovery (EOR), as well as by an established infrastructure for the capture, processing, and transportation of hazardous gases [1]. CO_2_ can be securely immobilized in geological formations through several well-recognized trapping mechanisms, including structural or stratigraphic trapping, residual capillary trapping, aqueous dissolution, and mineralization [2]. More recently, adsorption has gained attention as an increasingly relevant sequestration pathway, particularly in shale formations and coal seams where organic-rich matrices offer significant sorption capacity [2,3,4]. While these mechanisms operate concurrently, their relative contributions vary depending on the geochemical environment, petrophysical properties, depth, and in situ thermodynamic conditions of the storage formation. A key factor governing the fate of the CO_2_ plume is the timescale over which each trapping process evolves; dissolution, precipitation, and mineralization provide long-term security but occur slowly, whereas buoyancy-driven CO_2_ migration makes structural or stratigraphic trapping the dominant short-term mechanism.

In subsurface systems, low-permeability caprocks and faults act as primary structural seals that prevent upward CO_2_ migration. Although these barriers offer the most immediate form of storage security, they also exhibit the lowest long-term integrity among trapping options [5]. Traditional wellbore sealing agents such as cementitious materials, silicate gels, and epoxy resins are effective for plug-and-abandonment operations but are limited in their ability to penetrate deep reservoir environments. Consequently, crosslinked polymer-based gels have been extensively explored for conformance control and reservoir profiling, drawing from their successful applications in enhanced recovery [6,7,8]. While high-density polymer sealants can block preferential flow channels and restrict CO_2_ mobility [9], the prevailing research emphasis has focused primarily on mechanical sealing performance, including rheology, strength, and injectivity.

However, an underexplored yet potentially transformative aspect of polymer sealants is their capacity for CO_2_ adsorption, which could provide an additional in situ trapping pathway when these materials occupy cracks, microfractures, high-permeability streaks, or unconformities within the reservoir. Traditional plugging agents like cement and resins lack the ability for sorption; the physicochemical adsorption behaviour of CO_2_ on such deep-profile sealing materials has seldom been investigated. Understanding these molecular-scale interactions can serve as an important selection criterion for evaluating candidate sealing agents, enabling the improved prediction of long-term storage performance.

To address this gap, the present molecular simulation study investigates the adsorptive characteristics of CO_2_ under subsurface conditions onto a composite sealant comprising an interconnected network of amphiphilic cellulose-based biopolymer and pre-crosslinked polyacrylamide. By elucidating the adsorption behaviour at reservoir-relevant salinity, pressure and temperatures, this work provides new insights into the dual-function potential of polymer-based gels for conformance control and enhanced CO_2_ immobilization.

## 2. Results and Discussion

### 2.1. Interpenetrating Network Characterization

#### 2.1.1. Scanning Electron Microscope

**Figure 1:**
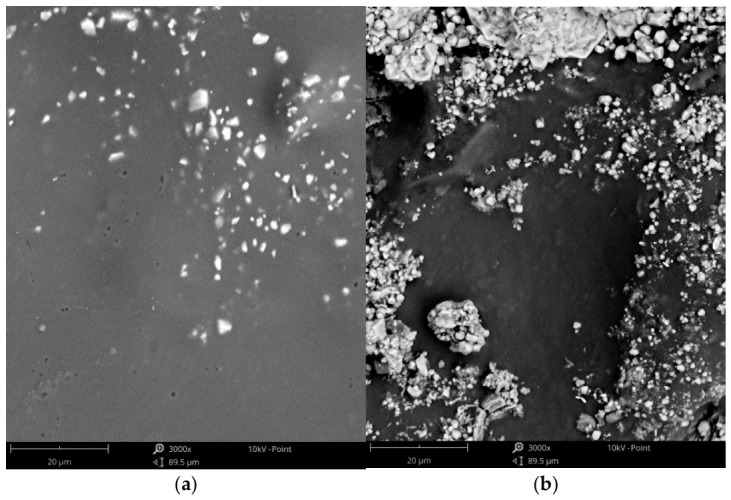
Scanning electron microscope image of composite gel particles D-I-AM-MBA-G-Cl (**left**, (**a**)) and D-II-AM-MBA-G-Cl (**right**, (**b**)) showing the contrast in topographic morphology; E_O_ = 10 KeV, 20 µm, 3000×.

The scanning electron microscope images of the interpenetrating network composite gel particles of D-I-AM-MBA-G-Cl and D-II-AM-MBA-G-Cl are presented in Figure 1. Here, it can be observed how the cellulose-based polymeric surfactants’ particles are embedded into the background polyacrylamide matrix. The hydrophobically modified cellulose derivatives appear as impregnations within the smooth texture of the pre-crosslinked polyacrylamide, forming a co-continuous phase. The surface morphology resembles an interparticle co-grafting between the synthetic polymer and the biobased amphiphilic polymer. From the SEM images, it is evident that D-II-AM-MBA-G-Cl attained more surface grafting than D-I-AM-MBA-G-Cl: the surface of the former appears rougher than that of the latter. The better interaction between methylene bisacrylamide crosslinked polyacrylamide and DPEA-Cell-OSO_3_^−^ II is also validated by the EDS elemental analyses presented in Table 1 and Table 2. The close elemental distribution of base elements carbon and nitrogen in terms of the atomic concentration and weight concentration proves a more uniform and homogeneous interaction across the material’s structure for D-II-AM-MBA-G-Cl compared to D-I-AM-MBA-G-Cl. The elemental analyses also revealed that the majority of the inorganic salt complexes and ionic content fragments are situated on the surface impregnations, while the organic portion of the IPN is embedded within the continuous matrix.

#### 2.1.2. Force Field Selection for Molecular Dynamics Simulation

The selection of the best-matched force field to simulate the molecular mechanics of the system is a very crucial step in this study. Here, the energy-minimization approach was adopted, wherein three prominent force fields commonly used for natural organic materials and biobased products were used to do a preliminary geometrical optimization of the structures. The COMPASS III force field gave the best output in terms of total energy reduction, as seen in Table 3, followed by DREIDING and then UNIVERSAL. Hence, COMPASS III was selected for the rest of the molecular dynamics simulations.

### 2.2. Molecular Dynamics Simulation Investigation

The sorption of CO_2_ onto sealant materials injected into the porous media of subsurface rocks before the gas injection provides a more sustainable pathway for geosequestration as compared to mere sealing of the leakage path. This is because gels and other types of plugging agents may have variable tendencies to fail after a very long time of continuous exposure to CO_2_ in acidic form, depending on the weight of the CO_2_ plume, the shear force, the harsh conditions of brine salinity and hardness, as well as the prevailing pressure and temperature. This is in line with the fact that different materials have a projected period of workability depending on the anticipated external stress [10]. If the sealants eventually give way mechanically, the sorption of CO_2_ into them will reasonably be the next line of preventive measure to keep the gas trapped. In addition, CO_2_-receptive gels are an augmentation to the natural adsorption mechanism of storage for reservoir rocks under prevailing temperature and pressure.

Speaking of interpenetrating network gels, there are several means by which CO_2_ can be trapped and sorbed into these networks. Firstly, sorption is dictated by the degree or density of the interpenetration of these networks, as well as the tightness or porosity. Here, channel paths within the interwoven network (which are as small as the size of a CO_2_ molecule) will form an effective barrier to the flow of CO_2_. Also, sorption is related to the presence of a CO_2_-philic functional group within the network. Here, the tendency for the gel to absorb CO_2_ into the water envelope at a certain thermodynamic state is a chemo-physical process.

#### 2.2.1. CO_2_ Adsorption Isotherm

**Figure 2:**
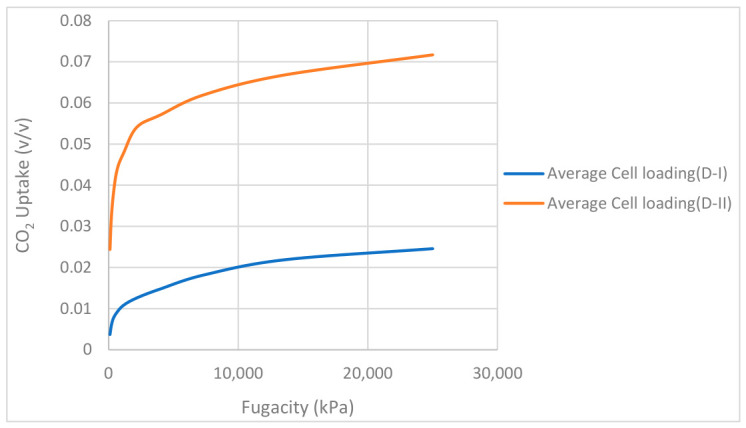
CO_2_ adsorption isotherm of interpenetrating polymer network materials of D-I-AM-MBA-G-Cl and D-II-AM-MBA-G-Cl at a temperature of 348.15 K.

In this study, both IPN gels D-I-AM-MBA-G-Cl and D-II-AM-MBA-G-Cl strongly adsorb CO_2,_ as seen in Figure 2, compared to single network polymer chains and mono-crosslinked gels [11,12,13,14]. As mentioned before, this superior sorption behaviour is linked to the mesh-like IPN constraint on the migration of CO_2_, which is not obtainable in regular polymer molecular architectures. D-II-AM-MBA-G-Cl has a better sorption performance compared to D-I-AM-MBA-G-Cl, characterized by a steep rise in the uptake of CO_2_ at lower pressures (Figure 2). DPEA-Cell-OSO_3_^−^ II biopolymeric material, one of the components of the D-II-AM-MBA-G-Cl interpenetrating network, is itself a porous material [10]. This implies that it has strong potential to trap CO_2_ within the matrix of its material, in addition to that of the crosslinked network; this explains its superior performance in the adsorption isotherm. This performance is also corroborated by a higher isosteric heat of adsorption of 9.53 kcal/mol at the investigated temperature, 348.15 K, compared to D-I-AM-MBA-G-Cl with a heat of adsorption of 4.87 kcal/mol. An adsorption process with free energy values of less than 10 kcal/mol has been recorded for composites containing amine-grafted polysaccharides [15].

#### 2.2.2. Sorption Sites

**Figure 3:**
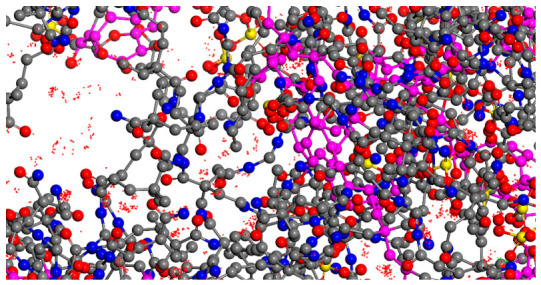
Sorption hotspots within the D-I-AM-MBA-G-Cl interpenetrating network are marked by red dots. The density of the dots gives a qualitative indication of the strength of adsorption at those points.

**Figure 4:**
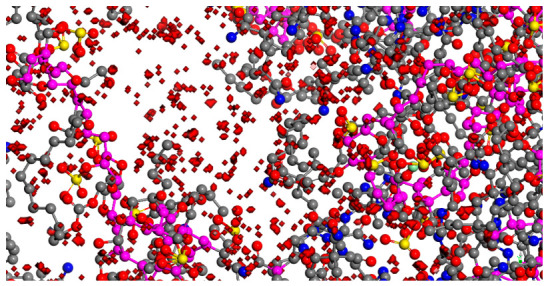
Sorption hotspots within the D-II-AM-MBA-G-Cl interpenetrating network are marked by red dots. The density of the dots gives a qualitative indication of the strength of adsorption at those points.

**Figure 5:**
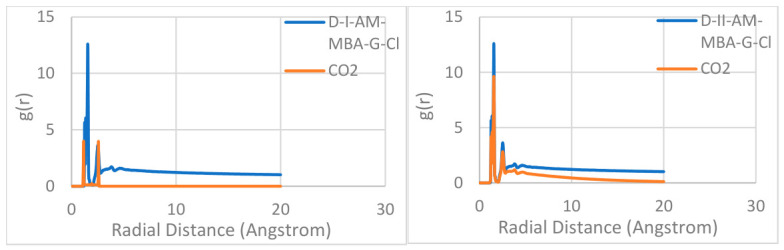
Radial distribution function of the interaction between CO_2_ and D-I-AM-MBA-G-Cl (**left**) and D-II-AM-MBA-G-Cl (**right**).

The sorption hotspots inside the IPN of the hybrid materials were analysed to further understand the adsorption nature using Figure 3 and Figure 4. Here, a portion of the distribution of CO_2_ molecules inside the interpenetrating polymer network matrix is presented. The red dots signify the clusters of CO_2_, where the density varies from region to region depending on the strength of sorption. A ball-and-stick display format was adopted, where the grey balls represent carbon, red balls represent oxygen, and blue balls represent the amine group, the alkyl group, and the anhydroglucose backbone on both the continuous acrylamide chain and cellulose chain, respectively, are represented in a magenta colour; the yellow functional group is the sulphate group. For both cases, it was discovered that the adsorption was physical in nature, as confirmed by the positive and low isosteric heat of adsorption. For D-I-AM-MBA-G-Cl, the CO_2_-philic points were discovered to be adjoining points on alkyl chains and the amine group; the ionic zone between the amine proton from the guanidine salt, the sulphate group, and the polyacrylamide backbone; the beta-reducing unit on the anhydroglucoside backbone; and the spaces between the alkyl chain and the carbonyl group on the carboxylate. There are also minor clusters of CO_2_ molecules on the amine group trapped inside the anhydroglucose rings. All these are depicted in Figure 3 with variable sizes of clusters. For D-II-AM-MBA-G-Cl, there are additional strong gathering points between the hydroxyl groups of the anhydroglucoside backbone, acrylamide fragment, and the alkyl chains. Also, there are clusters on the unsaturated alkoxy groups on the anhydroglucosidic chain, as represented in Figure 4. For both cases, these locations inside the interpenetrating network are crosspoints of overlay between the different chains and crosslinks. As such, CO_2_-philicity is not solely of specific functional groups, but pseudo-reactive points of attraction for CO_2_. Here, the different magnitudes of attractive and repulsive forces within the IPN architecture attain a balance for which the CO_2_ molecule attains a state of least energy. As seen in Figure 5, the radial distribution functions further prove a strong interaction of CO_2_ with the interpenetrating networks, as the proximities can be described as very close for both cases.

Traditional adsorbents like MFI Zeolites, mesoporous silica materials, and metal–organic frameworks (MOF) are cage-like 3-D structured systems commonly used in separation technology [16,17,18,19]. Points of sorption of particular sorbates in the network are pre-defined, based on the elemental composition and the porosity. On the other hand, crosslinked interpenetrating polymer chains are unconventional materials with a random network [20]. There are limitations to the prediction of the propagation of the interwoven structure; thus, sorption points are not premeditated. In the same vein, the sorption mechanisms are controlled by a complex combination of forces such as Van Der Waals, hydrophobicity–hydrophilicity and attractive–repulsive forces, hydrogen bonding, ionic complexation, polarity, steric interactions, and induced dipole interactions. However, this study provides support for the idea that that IPNs can be powerful adsorbents as well and can find usefulness in the subsurface capturing of CO_2_. Also, some investigations have revealed that cellulose and cellulose derivatives modified by the grafting of amine groups to the backbone are good adsorbents for water, harmful metal ions, oil, and dyes [21,22,23,24].

##### Binding Energies

The interaction energy coupled with the molecular orientation of CO_2_ at the sorption sites sheds further light on the manner and mechanism of adsorption within the IPN. A negative and low binding energy signifies adsorption, while a positive and high binding energy signifies the opposite. Also, the closer the interaction parameter χ is to zero, the stronger the communication between the sorbent site and the CO_2_ molecule. Thus, for the different hotspot configurations, the functional groups have variable interaction parameters. The negative binding energies confirmed adsorption for all molecular fragments, but this has variable strength, as depicted in Table 4. However, the interaction parameters signify those with the strongest influence on CO_2_. Of all those studied, the amine and the carbonyl groups had the best adsorption performance with negative chi values in the vicinity of 1 × 10^−2^. The binding energies are also very close to zero (Table 4). The architectural orientation of CO_2_ around these functional groups is such that it promotes associative interactions in which the carbon–carbon and oxygen–oxygen bonds are enhanced. In the case of the carbonyl group, the CO_2_ forms an encapsulating ring around it in a unique way. The regularity of the patterns in the periodic frames proves the consistency of communication between a sorbate and a sorbent.

The hydrophobic alkyl chain and the anhydroglucose unit of the cellulose sulphate backbone had the poorest adsorption performance, with the chi parameter being higher than 5. For the former, this reflects the fact that its pairing with CO_2_ molecules could not initiate any electrostatic interaction. Looking closely at the total potential energy, which is the summation of the Van Der Waals energy and electrostatic energy, the pairs that have the best attractive interactions are the ones with a reasonable balance of both attractive dispersive and coulombic forces. Since CO_2_ itself is a quadrupolar compound, any interface or space where it can be ionized will do well in trapping it. However, in this study, it was observed that the dispersive force has a greater influence on the outcome of CO_2_ binding within the matrix of the IPN architecture.

**Figure 6:**
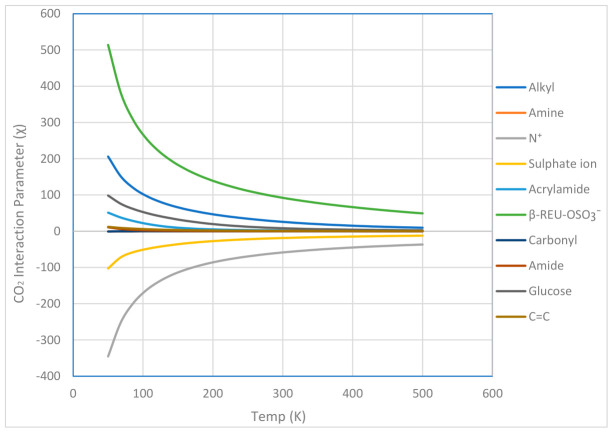
The influence of temperature on the interaction strength of CO_2_ and the different molecular fragments within the interpenetrating network.

Since the majority of the amine and the carbonyl groups are located on the crosslinks between acrylamide backbones and the inter-crosslink with the biopolymeric surfactant, a higher percentage of the adsorption clusters are located there. Hence, the crosslinking is critical to the adsorptive tendencies for CO_2,_ while the multifold nature of the interpenetrating network enhances it. The associative tendencies of the sorbent and sorbate, and the interaction energy, are further enhanced with temperature, as seen in Figure 6. As temperature increases, the interaction parameter χ approaches zero for all functional groups studied. This is because, at high temperatures, the sorbate and sorbent molecules have more kinetic energy to overcome the Arrhenius barrier and bind easily to create new pseudo ligands via bonding and non-bonding interactions such as hydrogen bonding, Van Der Waals’ forces, and electrostatic interactions. Nevertheless, the high-performance CO_2_ adsorbents such as the carbonyl group, the unsaturated alkoxy group, and the amine and acrylamide groups had good interaction parameters, even at low temperatures below ambient conditions.

Summing up the different interactions within the hotspots and correlating them with the CO_2_ density distribution inside the interpenetrating networks in Figure 3 and Figure 4, it could be inferred that the strongest sorption points within the IPN matrix are hotspots 7 and 2, respectively. The former is mainly the unsaturated covalent bonds along the chain of DPEA-Cell-OSO_3_^−^, while the latter is the ionic crosspoints between the sulphate and nitrogen protons and the amide group. Hotspot 2, in particular, shows that the strong adherence of CO_2_ is due to the balance of coulombic attraction with dispersive forces induced from the amide group, which contains both N-H stretch and the carbonyl group. Next in strength is hotspot 5, which also contains an amide group, but here it is in association with the glucose ring of the anhydroglucose unit. Though the interaction parameter is positive, it is relatively low, with a very low and negative binding energy. The presence of a saturated alkyl chain weakens the CO_2_ adsorption tendencies of hotspots 1, 4, and 6, despite the presence of nitrogen, sugar molecule, and the carbonyl group. The weakest hotspot in terms of CO_2_ retention is 3. This emphasizes that ionicity alone cannot effectively attract CO_2_. The conformation of the AGU chain could also contribute to the weak performance.

#### 2.2.3. Effect of Brine Salinity and Hardness on CO_2_ Adsorption

Connate water resident in reservoir rocks and geologic formations suitable for CO_2_ storage is characterized by moderate–high salt content; that is, it has a substantial amount of monovalent and divalent cations and anions. This, coupled with the high temperature, describes a typical subsurface scenario. Studies have shown that the saltiness of brine is a strong factor for the adsorption of chemical and other materials injected into the subsurface porous media for purposes like improved oil recovery [25,26]. Likewise, the brine content can strongly affect the CO_2_ adsorption and the interdependency on other sequestration mechanisms, thereby dictating the total amount of CO_2_ stored in an injection scheme [2,27].

**Figure 7:**
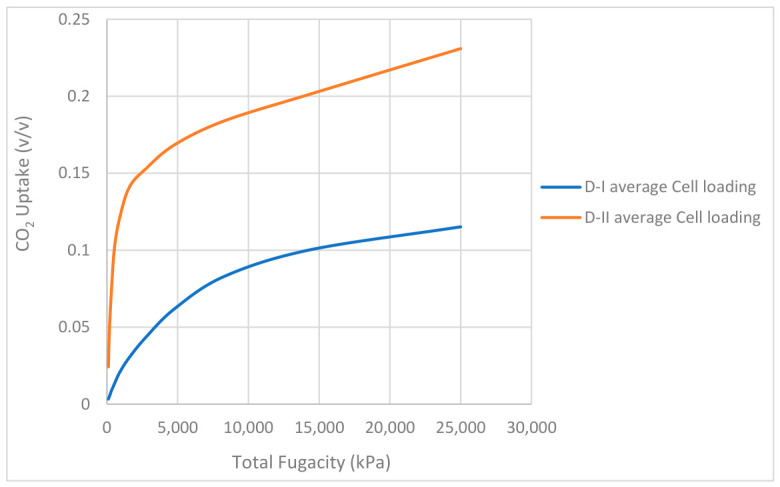
CO_2_ adsorption isotherm of interpenetrating polymer network materials of D-I-AM-MBA-G-Cl and D-II-AM-MBA-G-Cl in a brine environment at a temperature of 348.15 K.

In this study, salinity and brine hardness enhanced the adsorption of CO_2_ onto the IPNs D-I-AM-MBA-G-Cl and D-II-AM-MBA-G-Cl in a multifold way compared to the ordinary case without salt, but it was attained at the same temperature. As seen in Figure 7 for both cases, there was a rapid rise in the rate of adsorption within the first thousand fugacity, although D-II-AM-MBA-G-Cl had a better performance with a much steeper profile. This is characterized by the quick occupation of CO_2_-philic sites until they are saturated, after which the rate of adsorption becomes slower because of competition between molecules of CO_2_ to fill the few remaining adsorption sites.

The better sorption performance under brine conditions can be explained by an ionic steric effect within the interpenetrating network architecture. Here, the presence of cations and anions within the molecular space causes further intertwining of the strands along the backbones of the polymeric chains, thereby bringing the CO_2_-philic functional group hybrids closer. This way, the CO_2_-receptive hotspots are able to attract and retain more CO_2_ molecules. Ionic polymeric chains and polyelectrolytes can be observed to fold and assume a conformation with a smaller radius of gyration when exposed to an ionic environment [28,29]. This folding can promote entanglement in some portions of the network. Besides the proposed enhanced CO_2_-philicity due to ion-mediated steric hindrances, the nano displacement of molecular fragments creates a tighter pathway for the diffusion and dispersion of CO_2_ molecules within the network. This is particularly noticeable for the case of D-II-AM-MBA-G-Cl. After running a dynamics simulation for 5 nanoseconds to measure the mean square displacement of CO_2_ within the interpenetrating networks, the gas molecules had a diffusivity of 2 × 10^−6^ cm^2^/s for D-II-AM-MBA-G-Cl. This is way less than that of D-I-AM-MBA-G-Cl, at 4.87 × 10^−4^ cm^2^/s. This translates to CO_2_ molecules having less liberty for transport and displacement because of the instantaneous sorption for the former in relation to the latter.

##### Isotherm Fitting

**Figure 8:**
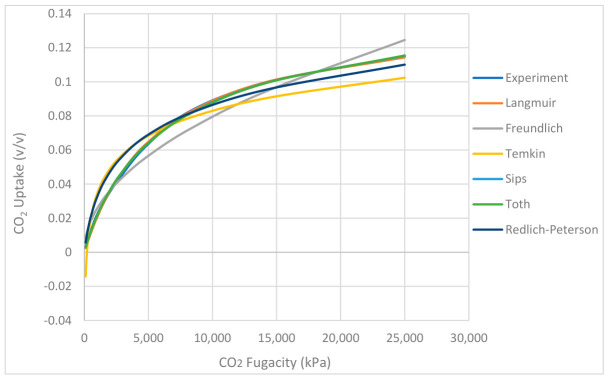
CO_2_ adsorption capacity matching of molecular dynamics simulation of the D-I-AM-MBA-G-Cl interpenetrating polymer network with isotherms. Langmuir R^2^ = 0.9991; Freundlich R^2^ = 0.9742; Temkin R^2^ = 0.9270; Sips R^2^ = 0.9996; Toth R^2^ = 0.9995; Redlich–Peterson R^2^ = 0.9654.

The adsorption of CO_2_ within the interpenetrating matrix of the salt-stabilized surface-active cellulose derivative and the crosslinked polyacrylamide at high temperatures in a saline environment is a complex process. To gain more insight, the adsorption profiles were compared to a series of selected established isotherms. Here, using nonlinear regression analysis, the simulated isotherms were matched to the Langmuir, Freundlich, Toth, Sips, Temkin, and Redlich–Peterson equations. As seen in Figure 8 for the case of D-I-AM-MBA-G-Cl, the best fitting was achieved for the Langmuir, Sips, and Toth isotherms, with R^2^ values higher than 0.99. The Sips equation best describes the adsorption behaviour with an R^2^ value of 0.9996. This characterizes the heterogeneity of the adsorption, as the adsorption energy and intensity vary over the active sites within the matrix network [27,30,31]. A heterogeneity exponent factor of 0.9203 (less than unity) suggests monolayer adsorption [32]. While this was deduced at a temperature of 348.15 K, a similar study on CO_2_ adsorption onto amine-modified activated carbon, which is also characterized by the Sips isotherm, suggests that the heterogeneity exponent is strongly dependent on temperature [31]. Here, at lesser temperatures of 333 K, 318 K, and 303 K, the heterogeneity factor gave values of 0.98, 1.04, and 1.07, respectively. This implies that, as the temperature reduces, adsorption takes a multilayer form inside the matrix of the ammonia-impregnated activated carbon. In general, the Sips isotherm, which is a hybrid of Langmuir and Freundlich, is known to be able to model the adsorption behaviour in media with irregular structures and undefined surface area, as we have in interpenetrating polymer networks. As seen in Figure 8, the adsorption behaviour is pressure-dependent as it tends towards saturation limits at high fugacity [33]. While the adsorption capacity is below 0.15 *v*/*v* of the cell, it can be inferred from the isotherm that the D-I-AM-MBA-G-Cl IPN will be suitable for selective gas adsorption since the material is functionalized chemically [31].

**Figure 9:**
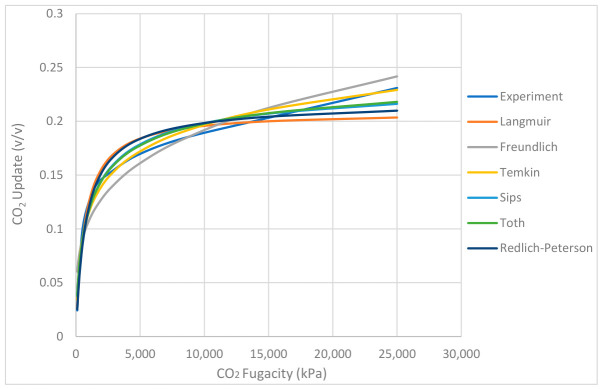
CO_2_ adsorption capacity matching of molecular dynamics simulation of the D-II-AM-MBA-G-Cl interpenetrating polymer network with isotherms. Langmuir R^2^ = 0.9651; Freundlich R^2^ = 0.9281; Temkin R^2^ = 0.9885; Sips R^2^ = 0.9796; Toth R^2^ = 0.9832; Redlich–Peterson R^2^ = 0.9681.

In the case of D-II-AM-MBA-G-Cl (Figure 9), the best-fitted isotherms are Temkin with a regression coefficient of 0.9885, and Toth with 0.9832. Both isotherms characterize the adsorption of CO_2_ within the composite matrix as a heterogeneous and multilayered sorption [34,35]. Ng et al. [30] stated that there are materials with homogeneous adsorption clusters within heterogeneous surfaces. This best describes the sorption sites inside the interpenetrating networks of both D-I-AM-MBA-G-Cl and D-II-AM-MBA-G-Cl. Since the pseudo-functionalized regions with varying adsorption energy that strongly attract CO_2_ molecules are situated within a network of repeating monomers, this gives the sorption sequence a segmented and recognizable pattern. Also, at times, the nature of the physicochemical interaction that takes place at an active site can create multilayer adsorption. This is because the attachment of the adsorbate to the surface creates pseudo-radicals therein for further sorption. Multilayered, heterogeneous CO_2_ adsorption has been reported in the literature for polysaccharides impregnated with amine functional groups [15,36]. The particular close match to the Temkin isotherm suggests the possibility of both adsorption and desorption occurring concurrently at the outer layers. Here, the heat of adsorption of the layered molecules decreases linearly with increasing surface coverage [37].

Generally, the information provided in Table 5 buttresses the fact that D-II-AM-MBA-G-Cl has a better CO_2_ adsorption capacity than D-I-AM-MBA-G-Cl. The Q_max_ parameter for all isotherm type-models, which denotes the ultimate capacity of a material to adsorb a species [38], CO_2_ in this case, is higher for D-II. The *n* parameter in the Freundlich isotherm is an empirical factor that describes the favourability of adsorption taking place in a medium [39]. Here, values of 0 to 1 denote weak adsorption, values of 1 to 2 indicate moderate adsorption, and values of 2 to 10 indicate good adsorption. Again, D-II has a value of 3.9684 while D-I has 2.0425. D-II-AM-MBA-G-Cl, having a Toth parameter, t, that deviates farther from unity at a value of 0.5431 compared to D-I-AM-MBA-G-Cl of 0.8371, implies that the adsorption is more heterogeneous in the former. This is corroborated by the SEM diagram (Figure 1), which shows the roughness of the topology. A heterogeneous surface of an adsorber often connotes that the material can entrap things that come into contact with it in more than one way [40]. The variety of adsorption mechanisms available is what makes a good adsorption material compared to a smooth homogeneous topology with a monotonic adsorption behaviour.

## 3. Conclusions

The theme of this study is the use of polymer-based interpenetrating network composite gels for the subsurface adsorption of CO_2,_ which has an extra sequestration mechanism in addition to conformance control via in situ plugging. Here, the adsorptive capability of synthetic polymer–biopolymer hybrid networks was investigated using molecular dynamics simulation. Novel biobased amphiphilic IPN materials, D-I-AM-MBA-G-Cl and D-II-AM-MBA-G-Cl, demonstrated reasonable adsorption. Though the IPN gels are unconventional adsorbents, unlike structured frameworks such as MOF, they can still adsorb CO_2_ due to the dense crosslinking and chain interpenetration. The strong adsorption was facilitated by the creation of sorption hotspots within the network with enhanced reception for CO_2_. The important functional groups within the sorption sites are the carbonyl groups, the amine groups, and the unsaturated alkoxy groups. The adsorption capacity was enhanced in saline conditions because of the ionic shielding effect, which makes the path for CO_2_ diffusion narrower. The adsorption of D-I-AM-MBA-G-Cl closely matches that of Langmuir, Sips, and Toth isotherms, while the best fits for D-II-AM-MBA-G-Cl are the Toth and Temkin isotherms. Overall, this study reveals that carefully designed plugging materials with adsorption capacity, in addition to elasto-adhesive properties, can positively enrich the selection process of agents for CO_2_ sequestration in geological formations. Future studies will look into the in situ adsorption of single-stream CO_2_ and multi-stream gas in media pre-saturated with the IPN gels. In these investigations, the adsorptive capacity will be considered as an extra conformance control performance indicator in addition to permeability reduction by sealing.

## 4. Materials and Methods

### 4.1. Formulation and Characterization

The chemicals used for the formulation of the interpenetrating polymer composites were as follows: guanidine hydrochloride; sodium hydroxide pellets; hydrophobically modified cellulose sulphates DPEA-Cell-OSO_3_^−^ I and DPEA-Cell-OSO_3_^−^ II; N,N-methylene bisacrylamide; acrylamide; ammonium persulphate. All chemicals were of analytical grade and were purchased from R&M chemicals via Advance Altimas Sdn Bhd, Petaling Jaya, Selangor. The hydrophobically modified cellulose derivatives were synthesized according to Afolabi et al. 2022 [41].

The crosslinked composite sealant was prepared by mixing hydrophobically modified cellulose sulphate derivatives DPEA-Cell-OSO_3_^−^ I and DPEA-Cell-OSO_3_^−^ II, with acrylamide in a ratio of 2:7 g/g in de-ionized water. The inter-crosslinking was initiated by adding 100 ppm of ammonium persulphate as the catalyst and 200 ppm of methylene bisacrylamide as the crosslinker under oxygen purging conditions for 30 min before allowing the reaction to stay for another 4 h at 50 °C. The hydrogel was stabilized with a solution of 5 g of guanidine hydrochloride and 0.02 g of methylene bisacrylamide in 50 mL de-ionized water for another 12 h at 70 °C.

The synthesized hydrogel was oven-dried with air assistance at 80 °C for 24 h. The resultant flakes were washed thoroughly with a 50/50 wt% acetone–ethanol mixture before subjecting them to another round of drying at 50 °C for 48 h. An EDS-assisted scanning electron microscope, a Brand Phenom Model ProX instrument manufactured by Thermo Fisher Scientific, was employed to study the surface morphology and elemental distribution.

### 4.2. Molecular Dynamics Simulation

**Figure 10:**
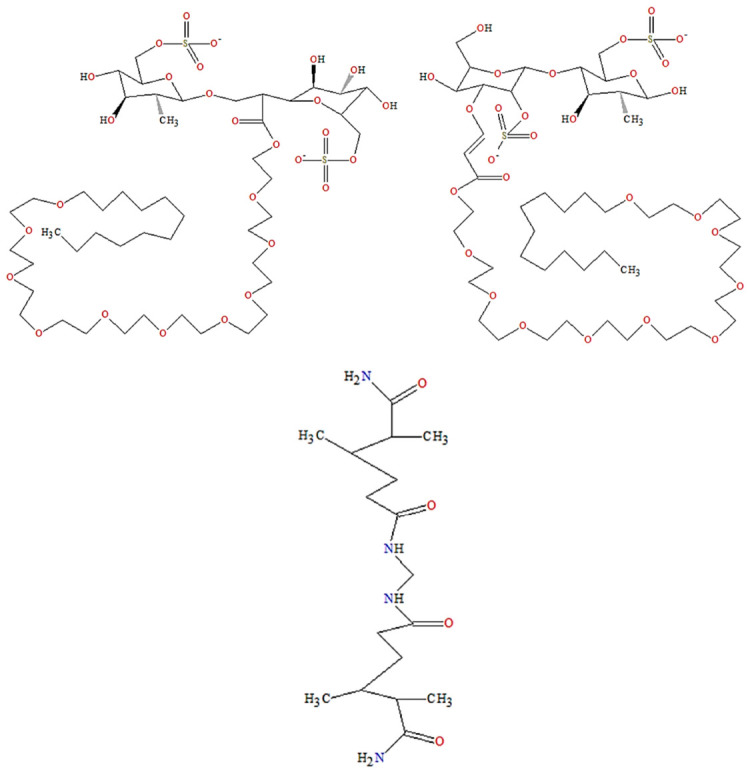
Monomers of DPEA-Cell-OSO_3_^−^ I (D-I), DPEA-Cell-OSO_3_^−^ II (D-II), and polyacrylamide crosslinked by methylene bisacrylamide, which make up the interpenetrating network.

The subjects of investigation in this study were interpenetrating polymer networks (IPNs) comprising hydrophobically modified cellulose sulphates DPEA-Cell-OSO_3_^−^ I (D-I) and DPEA-Cell-OSO_3_^−^ II (D-II) [41,42], and polyacrylamide crosslinked by N,N-methylene bisacrylamide. In this model, the amphiphilic cellulose derivatives were stabilized with guanidine hydrochloride. Figure 10 depicts the monomers that make up the semi-crosslinked interpenetrating networks, wherein there are separate IPNs for D-I and D-II.

The molecular architecture of the repeating units was relaxed energy-wise by subjecting them to geometry optimization using the COMPASS III, DREIDING, and UNIVERSAL force fields. Here, the Smart algorithm, with 500 iteration steps, was used to complete the task. A 0.00002 kcal/mol energy convergence tolerance was employed to enforce a quality for the molecular optimization. For non-bonded interactions, a cubic spline of 18.5 Å cut-off distance was used for the truncation. The force field with the best energy-reduction performance was selected for the rest of the simulation study. The “relaxed” versions of the monomers were used to build the polymers with a repeating unit of 15 monomers per polymer chain, subjected to further geometrical and energy minimization using the above criteria.

The interpenetrating polymer networks were designed by loading onto a cubic amorphous cell, with 5 chains each of the optimized polymers of the pre-crosslinked polyacrylamide, DPEA-Cell-OSO_3_^−^ I and DPEA-Cell-OSO_3_^−^ II. Also, 4 units of guanidine salt were loaded into the cells (size, 54.5 × 54.3 × 54.3 Angstrom), with a final average density of 0.73 g/cc. The cells were relaxed by running a geometry optimization and an atomistic dynamics simulation of 5000 ps at a temperature of 348.15 K, under a constant volume and constant number of molecules ensembled (NVT). Here, an atom-based summation method was used for the Van Der Waals forces, and Ewald summation was conducted for the electrostatic forces. Most subsurface geo-storage sites, where gels can be effectively deployed for sealing purposes, have a temperature range of 333 K to 408 K [43]. In this study, 348.15 K was particularly selected to evaluate the resilience of the novel IPN materials since the temperature marks the onset of harsh conditions.

To study the diffusivity and adsorptive interaction of CO_2_ with the IPN, CO_2_ was loaded onto the amorphous cell using the Metropolis Monte Carlo method under the NVE ensemble. Using the SORPTION^®^ module, the adsorption isotherm was studied by using 10 fugacity steps from ambient conditions (101.35 kPa) to 25,000 kPa. Here, 1 × 10^6^ production steps were run, with 1 × 10^5^ equilibration steps for each. The Metropolis Monte Carlo parameters are briefly stated in Table 6. CO_2_ uptake is calculated at every fixed pressure, then to the next step, which is the new pressure point in the isotherm simulation. The uptake is reported as the ratio of the volume concentration (*v*/*v*) of CO_2_ particles retained inside the interpenetrating polymer networks defined by the volume of the amorphous cell at the particular fugacity and reference temperature. For each fugacity, the isosteric heat is calculated using the expression below, and averaged over the entire pressure range of the study.


(1) $$Q=h_{{CO}_{2}}-h_{IPN}$$

Equation (1), representing the isosteric heat of adsorption, is also defined as the difference between the partial molar enthalpy of the sorbate; this is carbon dioxide gas and the interpenetrating network matrix. In terms of the Clapeyron equation, it can be rewritten as:


(2) $$Q=(\nu_{{CO}_{2}}-\nu_{IPN})\left[\frac{dp}{d(\ln T)}\right]=RT\left[\frac{d(\ln p)}{d(\ln T)}\right]$$

This shows the isosteric heat of adsorption in terms of the change in the partial molar volume from the sorbate reservoir to the interwoven polymer network cell as a function of the partial pressure derivative at the specific temperature of calculation.

For the saline condition case for each type of *IPN*, a ratio 3:1 monovalent to divalent ionic environment comprising sodium, calcium and chloride atoms was created, and the crosslinked interpenetrating cell was loaded into it in the molar ratio given in Table 7. To gain more insight into the adsorption behaviour and mechanism of CO_2_ within the *IPN* matrix of DPEA-Cell-OSO_3_^−^ I and DPEA-Cell-OSO_3_^−^ II with the methylene bisacrylamide crosslinked polyacrylamide, the uptake capacity was fitted with six standard isotherms: Langmuir, Freundlich, Toth, Temkin, Sips, and Redlich–Peterson. The fitting was done using nonlinear regression analysis, and the coefficient of determination, root mean square error, and the percentage of absolute relative error were used to evaluate the suitability of the isotherms.

The manner in which the CO_2_ molecule was adsorbed onto the interpenetrating network was investigated by calculating the binding energy and the interaction parameter, χ, at the adsorption site within the polymer matrix using Equations (3)–(5) below [44,45]. Here, the functional groups and/or the molecular fragments responsible for the attraction and retention of CO_2_ in the sorbate region were identified, and a base-screen configuration was used to design the sorbent–sorbate interaction. Around each sorption point, 100 frames of CO_2_ configuration with a 1 × 10^5^ cluster sampling size and 1 × 10^7^ energy sampling size were used to identify the lowest and most stable energy frames. These were, in turn, used to calculate the interaction energy for each base-screen pair in the presence of nearest fragments within the interpenetrating network.

(3) $$E_{binding}=E_{total}-(E_{sorbent}+E_{sorbate})$$

(4) $$E_{mix}=\frac{1}{2}zE_{binding}$$

(5) $$\chi =\frac{E_{mix}}{RT}$$

where *E_binding_* is the estimated binding energy at the adsorption site, *E_sorbent_* and *E_sorbate_* are the sorbent energy (IPN matrix) and sorbate energy (CO_2_), respectively, while *E_total_* is the total energy of the system. χ is the chi parameter, a dimensionless parameter for characterizing the association of multiple components. The *E_mix_* is the energy of mixing, another interaction parameter for describing the mixability of species. *R* is the universal gas constant, while *T* is the standard temperature. *z* is the lattice coordination number.

## Figures and Tables

**Table 1:** Energy-dispersive X-ray elemental distribution on D-I-AM-MBA-G-Cl flake (Figure 1a).

Element Name	Atomic Conc. (%)	Weight Conc. (%)
Nitrogen	40.32	38.74
Carbon	32.58	25.36
Oxygen	17.37	17.41
Chlorine	5.33	13.96
Sulphur	4.40	4.53

**Table 2:** Energy-dispersive X-ray elemental distribution on D-II-AM-MBA-G-Cl flake (Figure 1b).

Element Name	Atomic Conc. (%)	Weight Conc. (%)
Nitrogen	33.24	28.12
Carbon	32.19	25.86
Oxygen	17.11	17.56
Chlorine	12.57	17.41
Sulphur	4.89	11.05

**Table 3:** Energy minimization performances of DREIDING, UNIVERSAL, and COMPASS III for D-I and D-II.

	DPEA-Cell-OSO_3_^−^ I		DPEA-Cell-OSO_3_^−^ II	
Force Field	Initial Structure (kcal/mol)	Final Structure (kcal/mol)	Initial Structure (kcal/mol)	Final Structure (kcal/mol)
DREIDING	4.4 × 10^17^	103.03	1.5 × 10^15^	113.46
UNIVERSAL	2.1 × 10^17^	400.89	1.3 × 10^15^	321.16
COMPASS III	6.6 × 10^12^	2.20	2 × 10^11^	13.07

**Table 4:** The manner of interactions and the corresponding interaction parameters, binding energies, and potential energies of CO_2_ with different molecular fragments at the sorption sites within the interpenetrating polymer network.

CO_2_ Sorption Hotspots	Molecular Fragments’ Lowest Energy Configuration	Interaction Parameter at 348.15 K	Binding Energy at 348.15 K (Kcal/mol)	Average Binding Energy (Kcal/mol)	Average Van Der Waals Energy (Kcal/mol)	Average Electrostatic Energy (Kcal/mol)
Hotspot 1 (alkyl chain and amine group)	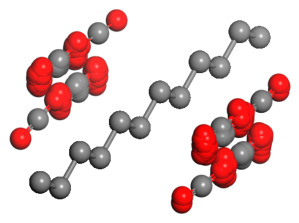	19.85	−1.23	−1.10	−1.87	
	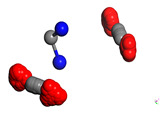	−0.01	−0.47	−0.45	−0.78	−0.04
Hotspot 2 (Nitrogen proton, sulphate ion and acrylamide chain)	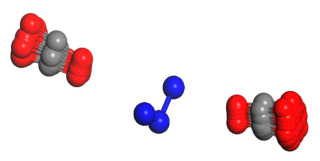	−50.93	−0.49	−0.39	−0.41	−0.82
	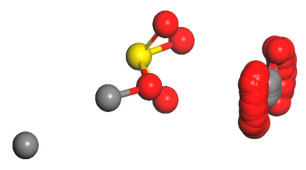	−16.44	−0.94	−0.72	−1.47	−0.62
	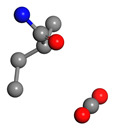	1.68	−0.81	−0.72	−1.53	−0.10
Hotspot 3 (β-REU with sulphate ion)	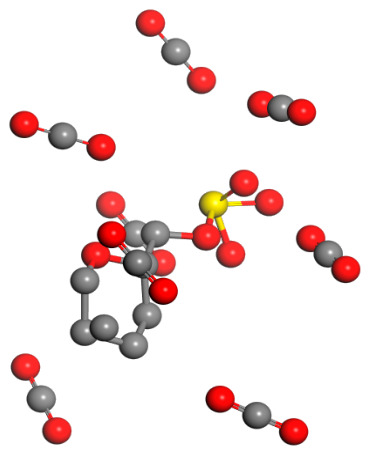	78.36	−1.33	−0.94	−1.86	−0.96
Hotspot 4 (alkyl chain and carbonyl group)	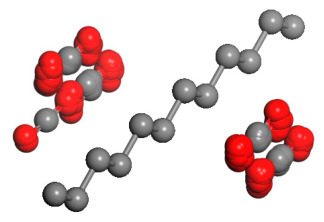	19.91	−1.23	−1.10	−1.87	
	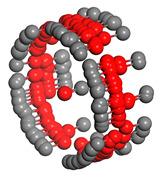	−0.01	−0.43	−0.42	−0.58	−0.08
Hotspot 5 (amide group and the glucose molecule)	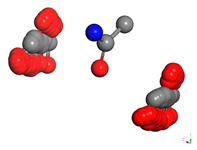	0.21	−0.58	−0.54	−0.95	−0.10
	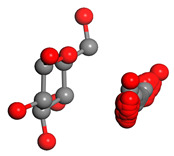	5.88	−1.02	−0.84	−2.12	−0.09
Hotspot 6 (Glucose molecule, amide group and alkyl chain)	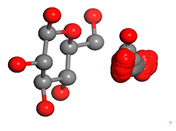	5.88	−1.02	−0.84	−2.11	−0.10
	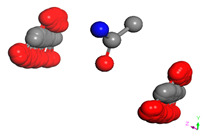	0.21	−0.59	−0.54	−0.94	−0.11
	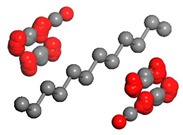	19.91	−1.23	−1.10	−1.87	
Hotspot 7 (unsaturated carbon–carbon double bond)	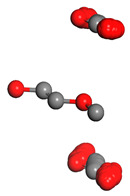	0.81	−0.71	−0.65	−1.21	−0.09

**Table 5:** Equilibrium fitting parameters for D-I-AM-MBA-G-Cl and D-II-AM-MBA-G-Cl.

Model	Equilibrium Parameters	D-I-AM-MBA-G-Cl	D-II-AM-MBA-G-Cl
Langmuir	Q_max_ (*v*/*v*)	0.1414	0.2091
	b (1/kPa)	0.0002	0.0015
	R^2^	0.9991	0.9651
	RMSE (*v*/*v*)	0.0012	0.0116
	ARE%	8.5961	7.6683
Freundlich	K_f_ ((*v*/*v*)·kPa^−1/n^)	0.0009	0.0188
	n	2.0425	3.9684
	R^2^	0.9742	0.9281
	RMSE (*v*/*v*)	0.0061	0.0167
	ARE%	40.5459	24.0222
Temkin	A (1/(*v*/*v*))	0.0051	0.0256
	B (RT/b)	0.0212	0.0355
	R^2^	0.9270	0.9885
	RMSE (*v*/*v*)	0.0103	0.0067
	ARE%	82.3102	7.7156
Sips	K (1/kPa)	0.0001	0.0009
	Qm (*v*/*v*)	0.1514	0.2409
	n	0.9203	0.6948
	R^2^	0.9996	0.9796
	RMSE (*v*/*v*)	0.0008	0.0089
	ARE%	3.5773	10.2995
Toth	Qm (*v*/*v*)	0.1563	0.2568
	B (1/kPa)	0.0002	0.0032
	t	0.8371	0.5431
	R^2^	0.9995	0.9832
	RMSE (*v*/*v*)	0.0009	0.0081
	ARE%	5.1586	9.3859
Redlich–Peterson	K_RP_ (1/(*v*/*v*))	0.0001	0.0003
	a_RP_ (1/(*v*/*v*))	0.0035	0.0015
	g	0.8164	0.9844
	R^2^	0.9654	0.9681
	RMSE (*v*/*v*)	0.0071	0.0111
	ARE%	37.5747	8.1759

**Table 6:** Sorption Metropolis Monte Carlo parameters used for the study.

Parameter	Ratio	Probability
Exchange	2	0.39
Conformer	1	0.20
Rotate	1	0.20
Translate	1	0.20
Regrow	0.1	0.02

**Table 7:** The molar composition of the brine system containing the interpenetrating biopolymer–synthetic polymer network.

S/N	Molecule	Weight %
1	Polyacrylamide–Methylene Bisacrylamide	46.50
2	DPEA-Cell-OSO_3_^−^ I/DPEA-Cell-OSO_3_^−^ II	52.41
3	Guanidine Hydrochloride	0.42
4	Chloride ion	0.11
5	Sodium ion	0.07
6	Calcium ion	0.04

## Data Availability

Data will be made available from the authors upon reasonable request.
